# SARS and hospital priority setting: a qualitative case study and evaluation

**DOI:** 10.1186/1472-6963-4-36

**Published:** 2004-12-19

**Authors:** Jennifer AH Bell, Sylvia Hyland, Tania DePellegrin, Ross EG Upshur, Mark Bernstein, Douglas K Martin

**Affiliations:** 1 University of Toronto Joint Centre for Bioethics, University of Toronto, Toronto, Canada; 2 Department of Health Policy, Management and Evaluation, University of Toronto, Toronto, Canada; 3 Department of Public Health Sciences, University of Toronto, Toronto, Canada; 4 Department of Surgery, University of Toronto, Toronto, Canada; 5 Department of Family and Community Medicine, University of Toronto, Toronto, Canada; 6 Division of Neurosurgery, Toronto Western Hospital, Toronto, Canada; 7 Pharmacy, Toronto Western Hospital, Toronto, Canada; 8 Primary Care Research Unit, Sunnybrook and Women's College Health Sciences Centre, Toronto, Canada

## Abstract

**Background:**

Priority setting is one of the most difficult issues facing hospitals because of funding restrictions and changing patient need. A deadly communicable disease outbreak, such as the Severe Acute Respiratory Syndrome (SARS) in Toronto in 2003, amplifies the difficulties of hospital priority setting. The purpose of this study is to describe and evaluate priority setting in a hospital in response to SARS using the ethical framework 'accountability for reasonableness'.

**Methods:**

This study was conducted at a large tertiary hospital in Toronto, Canada. There were two data sources: 1) over 200 key documents (e.g. emails, bulletins), and 2) 35 interviews with key informants. Analysis used a modified thematic technique in three phases: open coding, axial coding, and evaluation.

**Results:**

Participants described the types of priority setting decisions, the decision making process and the reasoning used. Although the hospital leadership made an effort to meet the conditions of 'accountability for reasonableness', they acknowledged that the decision making was not ideal. We described good practices and opportunities for improvement.

**Conclusions:**

'Accountability for reasonableness' is a framework that can be used to guide fair priority setting in health care organizations, such as hospitals. In the midst of a crisis such as SARS where guidance is incomplete, consequences uncertain, and information constantly changing, where hour-by-hour decisions involve life and death, fairness is more important rather than less.

## Background

As of August 12, 2003 there were 438 probable and suspected cases of Severe Acute Respiratory Syndrome (SARS) in Canada – the majority located in Toronto. In Toronto there were forty-four SARS related deaths and over 100 health care workers contracted SARS, placing intense pressure on Toronto's public health and hospital system [1].

Due to both the importance of hospitals in any health system and the difficulties they face, improving priority setting (also known as rationing or resource allocation) within hospitals is crucial. Priority setting is one of the most difficult issues facing hospitals because of funding restrictions and changing patient need. A deadly communicable disease outbreak, such as SARS in Toronto in 2003, amplifies the difficulties of hospital priority setting.

Key goals of priority setting in any context are legitimacy and fairness. 'Accountability for reasonableness' is an explicit ethical framework for legitimate and fair priority setting in health care [2]. It is internationally recognized as a framework that can guide priority setting in health systems and their institutions [3-5]. According to 'accountability for reasonableness', an institution's priority setting may be considered fair if four conditions are met: *relevance*, *publicity*, *appeals*, and *enforcement *(see Table 1).

In 2003, the outbreak of SARS further challenged priority setting decision makers in Toronto hospitals, creating decision making difficulties in relation to both SARS and non-SARS patients. To what extent should the need for containment over-ride other important needs? To what extent should the need for quick decisions over-ride the need for legitimate and fair decision making?

Only a few studies have directly examined priority setting in hospitals – two focused on technology acquisition [6,7], one on strategic planning [8], one on a hospital drug formulary [9], and two on hospital ICUs [10,11]. The latter four studies used 'accountability for reasonableness' as the study framework. To our knowledge there have been no studies of hospital priority setting during an emergency response to a communicable disease outbreak.

The purpose of this study was to describe priority setting in a hospital in response to SARS and evaluate it using 'accountability for reasonableness'.

## Methods

### Design

To describe priority setting we used qualitative case study methods. A case study is "an empirical inquiry that investigates a contemporary phenomenon within its real-life context" [12]. This is an appropriate method because priority setting in hospitals is complex, context-dependent, and involves social processes. To evaluate the description we used the four conditions of 'accountability for reasonableness' (described in Table 1).

### Setting

This case study was conducted at a large tertiary hospital in Toronto, Canada.

### Sample

We sampled key documents and people. We used theoretical sampling to determine which people and documents were 'key'. Included among the individuals sampled were senior administrators, managers, physicians, nurses, patients and family members.

### Data collection

There were two sources of data: (1) over two hundred key documents (e.g. emails, minutes of meetings), and (2) 35 interviews with key informants (senior administrators (6), physicians (10), managers (5), nurses (5), a patient (1), family members (2), and other staff (6)). Interviews were audiotaped and transcribed. Interview participants were asked to describe priority setting decisions in relation to SARS and their thoughts about it. We developed an interview guide based on previous research and improved it through pilot interviews with personnel from other hospitals. As is traditional with qualitative studies, the interview guide was modified during the study to explore emerging themes.

### Data analysis

The data were analyzed concurrent with collection using a modified thematic approach in three phases: open coding, axial coding, and evaluation. In open coding, the data were fractured by identifying chunks of data that relate to a concept or idea. In axial coding, the concepts were organized under overarching themes (i.e. the four conditions of 'accountability for reasonableness'). In evaluation, the descriptive data were compared with the conditions of 'accountability for reasonableness' – correspondence with the framework was considered 'good practice'; instances where the conditions are not met were considered 'opportunities for improvement'. Concepts were formalized and made explicit through the writing of the findings [13].

The validity of the interpretations was enhanced in four ways [14]. First, the coding was conducted in collaboration between two researchers, thus limiting the influence of any one person's biases. Second, the coding was reviewed and modified by an interdisciplinary team who provided challenges that were resolved through consensus. Third, the findings were presented to participants who verified the findings – traditionally called a 'member check'. Finally, all research activities were rigorously documented to permit a critical appraisal of the methods [15].

### Research ethics

Approval for this project was obtained from the participating hospital's Research Ethics Board. Written informed consent was obtained from each individual before being interviewed. All data were kept confidential and viewed only by the research team. No individuals have been identified without their explicit agreement.

## Results

In this section we describe one hospital's priority setting in response to SARS by focusing on the types of decisions, the decision making process, and the supportive reasoning. We then evaluate our findings using the four conditions of 'accountability for reasonableness'. We have also included verbatim quotes from participants to illustrate key points.

### I. Description of priority setting

#### Types of priority setting decisions

There were two distinct phases of priority setting at the hospital during the SARS outbreak. First, during the initial days of the outbreak, decisions were made in order to contain the spread of the virus. The Ontario Ministry of Health and Long-Term Care (MOHLTC) directed Toronto acute care hospitals to establish or maintain as necessary a SARS isolation area, restrict patient visits, limit visitors and suspend selected patient transfers. Second, after the initial weeks of the outbreak, was a 'ramp up' phase during which the hospital gradually increased its level of activity. Throughout both phases, priority setting decisions can be organized according to four specific types: decisions relating to staff and patients, beds/rooms, clinical activity, and visitors.

##### 1) Staff and patients

Staff were allocated for SARS patients in the SARS unit and ICU, screening at the doors, manning the site command centre, or helping out in occupational health; pregnant and immunosuppressed staff were either redeployed to low-risk activities or sent home; staff deemed non-essential were sent home with pay; students were sent home and educational rounds were cancelled. General medicine patients were transferred to other medical units to maintain the SARS isolation unit; out-patients who had been waiting for non-emergency surgery or clinic appointments were told to wait indefinitely.

##### 2) Beds/rooms

The SARS isolation unit required negative pressure beds. The hospital created these spaces in a specific isolation unit on a general medicine floor, and in the ICU and Emergency. Admissions to negative pressure beds were decided case-by-case and were based on the assessment of the referring physician, the hospital's infectious disease representative, and the hospital intensivist.

##### 3) Clinical activity

All non-emergency surgery and ambulatory care were cancelled during the initial 7–10 days of SARS. During the ramp up phase, clinical activity volumes increased in percentages allowing for urgent cases to be seen as determined by physicians. Operating room time was allotted by division – each individual surgeon reported to their division head the cases they considered urgent. This activity was coordinated by the hospital's command center.

##### 4) Visitors

A 'no visitors' policy was enforced during the initial stages of SARS except for compassionate grounds as determined by the nurse manager or nurse in charge of the particular unit, in consultation with the attending physician and the hospital command centre. During the ramp up phase, the hospital relaxed the no visitor policy according to changing directives from the MOHLTC and the human resources available for screening at the doors.

#### Decision making process

Priority setting decisions were made across all levels of the institution. We identified four groups of key decision makers: Corporate Command, Hospital Command, Department Management/Chiefs, and Individual Clinicians. MOHLTC directives were interpreted by a team of senior administrators (corporate command) and then communicated to the hospital's command centre. The hospital's command centre implemented the recommendations from the corporate command in accordance with patient population requirements and physical logistics. Department managers and clinical staff also participated in allocating human resources and determining patient care priorities. The corporate command was in constant communication with the hospital's command centre who maintained communication with the managers and other leaders through teleconferencing and email.

#### Priority setting reasoning

**Figure 1:**
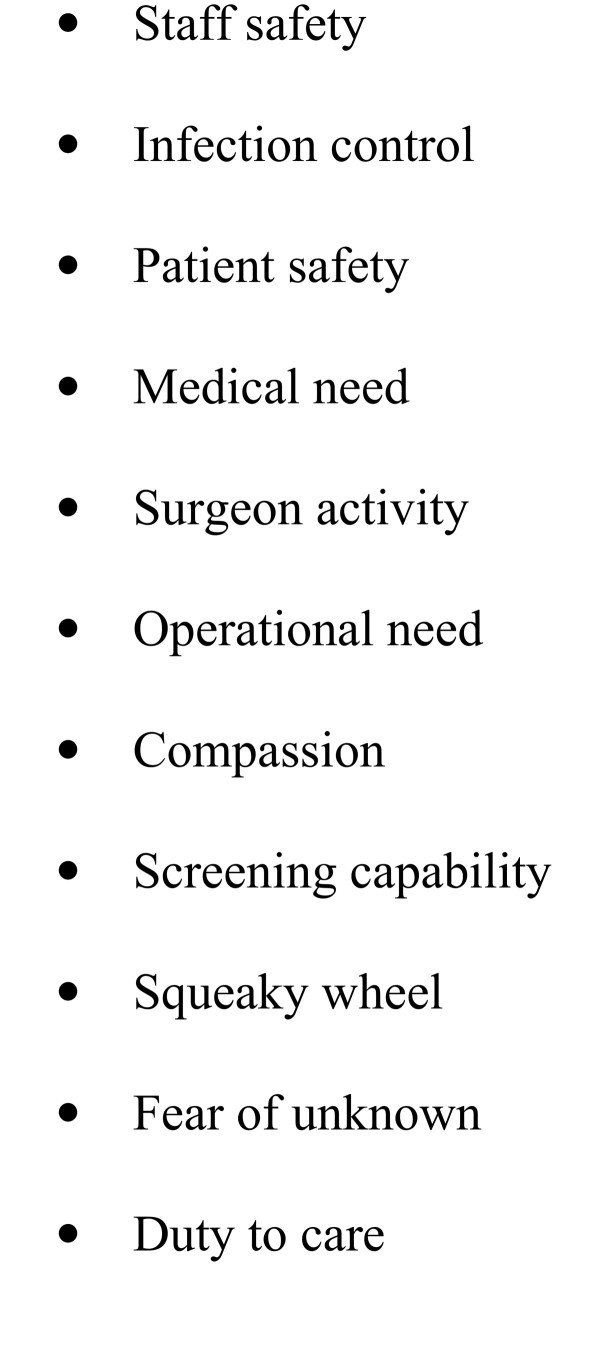
Reasons justifying priority setting decisions

Throughout each stage of the SARS outbreak, safety was the primary rationale underpinning priority setting. During the early stages of SARS, decisions were made for infection control focused on protecting staff. During the ramp up phase, decisions were based more on a duty to care for patients, emphasizing the hospital's role in the community. However, in addition, there were several other reasons used in support of each decision (See Figure 1).

During the ramp up phase the reasoning shifted to address patient need. Leaders recognized that the hospital could not operate under the shut down conditions for very long; urgent cases were quickly becoming emergent. Thus, though staff and patient safety remained a primary concern, very few of the decisions can be linked solely to safety; rather, decisions involved clusters of reasons. Table 2 describes decisions made, the reasons used, and the level at which they were made.

### II. Evaluation of priority setting using 'accountability for reasonableness'

#### 1) Relevance

Many participants confirmed that staff and patient safety was, appropriately, the primary criterion used in the decision making process.

"I think due to the fact that this was so communicable, and I think, certainly felt, most people felt this was all being done in our best interest."

Some expressed concern about the relevance of the reasoning used in the allocation of OR time and the visitor policy. For example, one surgeon commented that the OR schedule was allocated by division as opposed to being allocated by patient need. Similarly, the visitor policy was appreciated by family members of patients on an abstract level but some still could not understand why exceptions to the policy could not be made in certain instances.

Many participants found it difficult to evaluate the relevance of the reasons underlying many of the MOHLTC directives because the directives did not explicitly describe the reasoning involved.

#### 2) Publicity

Priority setting decisions and the reasons behind them were readily accessible to those directly involved in making the decisions. However, even though decisions and reasons were regularly distributed via email, or posted on the hospital intranet and world-wide web, many felt that communication beyond the core group of decision makers was incomplete. At the height of the crisis, MOHLTC directives were changing almost hourly, and this made real-time communications to the front lines difficult. It was generally felt that communication was excellent, with room for improvement.

"Even though we have a very good communications team you know some people are still left out of the loop – they don't read the paper they don't listen to the radio they don't read their emails or they don't have email. So, there are still small pockets of lack of or miscommunication, so communication is always something that we need to improve."

#### 3) Revision/appeals

There was no formal revisions/appeals mechanism. Instead the hospital CEO felt it was important to address all disagreements personally. Many participants thought there were ample opportunities for informal discussion and debate in meetings and email communication. However, one participant commented that without formal appeals mechanisms, some stakeholders participated unfairly by using a 'squeaky wheel' approach.

" [B]y appealing the process, the sickly squeaky wheel method of appeal, we just begged, pleaded, ranted, raved, called back, called back."

#### 4) Enforcement

Overwhelmingly, participants thought the process was as fair as it could have been given the time constraints and the knowledge base at the particular time. The hospital leadership made an effort to meeting the conditions of 'accountability for reasonableness'. However, some felt that the decision making was not ideal.

"At a moment of crisis which I think that SARS was, there's not always opportunity for full and open and even decision making."

Some stated that more support and accountability implementing decisions could have occurred – that there was a gap between the decisions that were made in high level administration and the implementation of those decisions at the front lines.

"Most of us felt, you know the decisions were made, up there, and we could understand them, we could agree with them, but we were the ones who had to live with them. And there was nobody who really came and asked us what that was like. There was some, it wasn't that there was nothing – but there wasn't a sense of being listened to the way that we needed to be listened to, the way that we needed to be supported."

Some participants expressed concern that many staff started relying on senior management to make many of the decisions for them.

"When you go into another mode that commands and controls, doesn't take too long until you understand how comfortable and actually how easy that is. It is way easier to do what you're told."

## Conclusions

This study examined priority setting at one Toronto hospital as it responded to the 2003 SARS crisis. Even though the crisis created safety concerns and time constraints that impinged upon decision making, this hospital endeavoured to meet the four conditions of 'accountability for reasonableness'.

By describing and evaluating priority setting using the four conditions of 'accountability for reasonableness', we are able to identify examples of 'good practices' that other hospital should emulate, and 'opportunities for improvement' that this and other hospitals should consider.

We identified the following *good practices*: 1) staff and patient safety was the primary criterion in decision making, but each decision was based on a cluster of relevant reasons – decision makers' use of clusters of relevant reasons has been identified and discussed in a previous study [16], 2) decisions were regularly accessible on hospital email, intranet and the world-wide web; 3) challenges were addressed directly by the CEO; 4) hospital leadership made a concerted effort to meet the conditions of 'accountability for reasonableness'. We also identified the following *opportunities for improvement*: 1) patients and families did not have access to the reasons for many decisions, including the visitation policy and ramp-up of clinical activities; 2) a formal revision/appeals mechanism could help improve the quality of decision making and alleviate the unfair reliance on the 'squeaky wheel' phenomenon; 3) OR time was allocated by division, rather than by patient need, and these decisions should be discussed more fully; 4) institutional leaders should maintain two-way contact with front-line staff who are implementing priority setting decisions – this will provide support and enhance accountability for decision making by staff.

'Accountability for reasonableness' is a framework that can be used to guide legitimate and fair priority setting in health care organizations, such as hospitals. It assumes that the time and effort required for meeting the conditions of fairness is justified for two reasons: First, it is important to act ethically and be perceived to be acting ethically – in this case, fairly. Second, acting ethically can help an organization achieve 'goodwill' benefits including, but not limited to, increased trust and satisfaction and decreased complaints. However, it is clear from this scope of decisions examined in this study that time constraints imposed on a health care organization by a highly communicable and potentially fatal infectious disease creates significant priority setting difficulties. It may appear that the conditions of 'accountability for reasonableness' are too demanding to implement in the time constraints – that perhaps containment should take precedence over procedural requirements. We disagree. During the SARS outbreak the hospital's leadership developed and implemented several sophisticated processes to help with their crisis management. Tailoring those processes to meet the four conditions of 'accountability for reasonableness' is not any more difficult or demanding. Moreover, we argue, and some of the participants also argued, that in the midst of a crisis where guidance is incomplete, consequences uncertain, and information constantly changing, where hour-by-hour decisions involve life and death, fairness is more important rather than less.

Our findings are limited in that they may not be generalizable to other hospitals. However, generalizability is not the goal of qualitative studies like this. We expect that other hospitals may benefit from the insights provided in this study. For example, this was the first time that 'accountability for reasonableness' has been used to evaluate priority setting in response to an infectious disease outbreak. Other hospitals in similar crises can use 'accountability for reasonableness' to help evaluate and enhance the fairness of their priority setting [17].

The best assurance of fair priority setting in a crisis is fair priority setting everyday. A health care organization that incorporates legitimate and fair decision making everyday, where the decision making culture of the organization is permeated with the conditions of 'accountability for reasonableness', will be primed to meet the challenges of fair priority setting in a crisis. This may be the most important lesson to take from this study.

## Competing interests

SH and MB are employees of the organization studied here.

## Authors' contributions

JB was primarily responsible for acquisition and analysis of data. All authors made substantial contributions to conception and design of the study, data analysis and interpretation, and have been involved in drafting the article or revising it critically for important intellectual content. All authors have given final approval of the version to be published.

## Pre-publication history

The pre-publication history for this paper can be accessed here:



## Figures and Tables

**Table 1:** The four conditions of 'accountability for reasonableness'

*Relevance*	Rationales for priority setting decisions must rest on reasons (evidence and principles) that 'fair-minded' people can agree are relevant in the context. 'Fair-minded people seek to cooperate according to terms they can justify to each other – this narrows, though does not eliminate, the scope of controversy, which is further narrowed by specifying that reasons must be relevant to the specific priority setting context.
*Publicity*	Priority setting decisions and their rationales must be publicly accessible.
*Revision/Appeals*	There must be a mechanism for challenge, including the opportunity for revising decisions in light of considerations that stakeholders may raise.
*Enforcement*	There is either voluntary or public regulation of the process to ensure that the first three conditions are met.

**Table 2:** List of decisions, reasons, and decision level

**Decisions: Staff and Patients**	**Reasons**	**Decision Level**
Determine which staff to deploy to help with screening at the doors	Operational need; Screening capability; Infection control; Medical need	Hospital Command
Determine urgent patients and care for those first	Medical need	Individual Clinicians
The hospital as a whole determined few hospital workers unessential	Operational need; Screening capability; Infection Control	Hospital Command
Redeploy staff from screening back to clinical areas	Medical need; Duty to care; Operational need	Hospital Command
Hire screeners	Medical need; Operational need; Infection control	Hospital Command
Remove pregnant staff from the clinical environment	Staff safety	Corporate Command; Hospital Command
Decant staff and inpatients (25) from 8^th ^floor general medicine to make room for SARS unit and potential SARS patients	Operational need; Medical need	Hospital Command; Department Managers/Chiefs
Separate staff entrance from visitor and patient entrance	Operational need; Infection control	Corporate Command; Hospital Command
Send staff home	Infection control	Department Managers/Chiefs
**Decisions: Beds**	**Reasons**	**Level Made At**
Accept SARS patient transfers from other hospitals	Duty to care	Corporate Command; Individual Clinicians
Each GTA and Simcoe County hospital to establish a SARS specific isolation unit.	Infection control	MOHLTC
Hospitals greater than 500 beds will be expected to provide a 30 bed unit each. (Mar 27)		
Create SARS unit physical space on 8B with negative pressure capabilities	Directive; Infection control; Medical need; Operational need; Duty to care	Hospital Command; Department Managers/Chiefs; Individual Clinicians
**Decisions: Clinical Activity**	**Reasons**	**Decision Level**
Maintain emergency based activity during initial days of outbreak	Duty to care; Medical need	Corporate Command; Hospital Command
Ramp up clinical activity	Duty to care; Medical need	Corporate Command
Allocate OR time by division	Medical need; Surgeon activity	Department Managers/Chiefs
Determine which patient needed urgent OR care this could be listed second	Medical need	Individual Clinicians
SARS II – the decision not to cancel surgery again	Medical need; Duty to care	Corporate Command
Treat some 'elective cases' in the OR as being urgent	Medical need; Surgeon activity; Duty to care; Squeaky wheel	Individual Clinicians; Department Managers/Chiefs
Determine what/who is emergent and urgent in terms of clinical volumes in family medicine	Screening capability; Medical need; Squeaky wheel	Department Managers/Chiefs; Individual Clinicians
Family Medicine did not go out into the community to provide care in the initial stages of SARS (care to detox centres, shelters)	Infection control; Screening capability	Corporate Command; Department Managers/Chiefs
**Decisions: Visitors**	**Reasons**	**Decision Level**
No Visitor Policy except for compassionate grounds (such as palliative care, critically ill children or visiting a patient whose death may be imminent)	Infection control	MOHLTC
Restrict visitors for certain hours (5–9 pm)	Screening capability	Hospital Command
Lift visiting restrictions on case-by-case basis	Compassion; Squeaky wheel; Medical need	Department Managers/Chiefs
Hospitals must restrict access to each hospital site. Ideally, access should be restricted to one staff and one public entrance for each building	Infection control	MOHLTC
